# *Myrtus communis* essential oil mitigates bisphenol A‐induced reproductive and lipidomic alterations in a male rat model

**DOI:** 10.14814/phy2.70628

**Published:** 2025-10-23

**Authors:** Mhimdi Mariem, Selmi Slimen, Sut Stefania, Peron Gregorio, Jridi Mourad, Dallacqua Stefano, Sebai Hichem

**Affiliations:** ^1^ Laboratory of Functional Physiology and Valorization of Bio‐Ressources Higher Institute of Biotechnology of Beja University of Jendouba Beja Tunisia; ^2^ Department of Pharmaceutical and Pharmacological Sciences University of Padova Padova Italy; ^3^ Department of Molecular and Translational Medicine (DMMT) University of Brescia Brescia Italy

**Keywords:** bisphenol A, endocrine disruptor, germ cell maturation, lipid metabolism, *Myrtus communis*, testicular steroid hormones

## Abstract

Bisphenol A (BPA), a widespread endocrine disruptor, induces male reproductive toxicity by impairing spermatogenesis and altering hormonal homeostasis. This study investigated the protective effects of *Myrtus communis* essential oil (EOMC) or vitamin E (Vit E) in adult male rats exposed to BPA (100 mg/kg/day). BPA administration led to testicular damage, decreased sperm quality, hormonal imbalance, oxidative stress, and changes in lipid metabolism. Vit E preserved seminiferous tubule structure and reduced the presence of immature germ cells, though partial disruption of spermatogenesis persisted. EOMC displayed dose‐dependent protective effects at 50, 100, and 200 mg/kg. At the highest dose, EOMC improved sperm parameters, restored testicular histology, preserved membrane phospholipid composition, and normalized levels of steroid hormones including testosterone, estradiol, progesterone, and cortisol. It also prevented body weight loss induced by BPA. Although Vit E provided better protection of tubule structure, EOMC at 200 mg/kg offered broader benefits across multiple reproductive parameters. These findings highlight the therapeutic potential of high‐dose EOMC in mitigating BPA‐induced male reproductive toxicity and support its use as a natural protective agent.

## 1 INTRODUCTION

In recent years, increasing scientific evidence highlighted the significant role of specific environmental pollutants that contribute to fertility problems (Srivastava & Gupta, 2018). Among these pollutants, bisphenol A (BPA) has emerged as a prevalent endocrine‐disrupting chemical (EDC), commonly found in epoxy resins, polycarbonate plastics, and electronic materials (Costa & Cairrao, 2024; Li et al., 2024). Due to its extensive use in consumer goods, environmental exposure to BPA raises substantial concerns for public health problems. Human exposure to BPA can occur through the use of plastic BPA‐containing food packaging, aerosols, and personal care products and can enter humans' bodies via ingestion, inhalation, and dermal absorption (Banerjee et al., 2024; Wang et al., 2024). Accordingly, it has been indicated that BPA was detectable in the urine of over 90% of the American population (Zhang et al., 2016). BPA exposure has been associated with a range of adverse effects on human and animal health, including disruptions of metabolism (Fenichel et al., 2013), neurodevelopment (Welch & Mulligan, 2022), behavior (Palanza et al., 2008), immune (Yang et al., 2015), cardiovascular (Fonseca et al., 2022), and reproductive systems (Yadav et al., 2023). Mechanistically, BPA toxicity in the male reproductive system has been related to the affinity for estrogen receptors (ER), particularly ERα, ERβ, and estrogen‐related receptor gamma (ERR‐γ), resulting in reduced testosterone levels, detrimental impacts on hormonal balance, and impaired semen parameters (Norazit et al., 2012; Shamhari et al., 2021).

Recent findings suggested that the sperm plasma membrane plays a crucial role in maintaining sperm functionality (Du et al., 2016; Lenzi et al., 1996; Shan et al., 2021). It is composed of phospholipids, sterols, and glycolipids, with phospholipids, especially those rich in polyunsaturated fatty acids (PUFAs), providing essential membrane fluidity. The lipid composition of the sperm membrane is strictly related to cell motility, viability, and resistance to environmental stressors, particularly to oxidative stress damage and lipid peroxidation (Stanishevskaya et al., 2023). A previous study demonstrated that BPA exposure, together with the altered spermatogenesis related to hormonal derangements, was associated with direct sperm cell damage by triggering oxidative stress, lipid peroxidation, impaired sperm motility, morphological changes, and decreased adenosine triphosphate (ATP) levels in an animal model (Grami et al., 2020).

Essential oils (EOs) are natural products extracted from plants and are well‐known for their diverse biological activities and therapeutic applications. Plant‐derived EOs are frequently used in pharmaceuticals for their antimicrobial, antioxidant (Cherrat et al., 2014; Mir, 2024), antidiabetic, anticancer, and anti‐inflammatory properties (Belahcene et al., 2023; Elshafie & Camele, 2017). EOMC is rich in bioactive compounds that exhibit a wide spectrum of biological effects. Additionally, antioxidants, such as Vit E, are potent protectors of cell membranes from oxidative damage and exhibit cholesterol‐lowering properties (Peña‐Corona et al., 2023). In humans, Vit E has been used as an empirical treatment to counteract oxidative stress, improve sperm parameters, and increase live birth rates in assisted reproduction techniques (ART) (Zhou et al., 2022).

Despite the demonstrated benefits of antioxidants like Vit E, there is limited research on the impact of EOMC essential oil on male fertility. This study aimed to investigate the potential protective effects of EOMC against BPA‐induced reproductive toxicity in male Wistar rats and to elucidate the underlying mechanisms involved.

## 2 MATERIALS AND METHODS

### 2.1 Plant collection and essential oils preparation

Myrtle leaves were harvested in March in the Hammam Bourguiba region (north‐west of Tunisia) and identified by a trained and experienced botanist (C.H.). The leaves of *Myrtus communis* were submitted to hydro distillation for 3 h using Clevenger type apparatus. Briefly, the plant was immersed in water and heated to boiling, after which the essential oils were evaporated together with water vapor and finally collected in a condenser. The distillate was isolated and dried over anhydrous sodium sulfate. To ensure reproducibility, the extraction process was performed in triplicate independent preparations. The oil fractions were stored at 4°C until use (El Hartiti et al., 2020).

### 2.2 In vitro antioxidant activities assays

The assessment of antioxidant efficacy of EOMC at various concentrations was conducted through two in vitro assays, the determination of reducing power and the β‐Carotene bleaching test. All experiments were executed in triplicate.

The capacity of EOMC to reduce iron was assessed following the protocol of Yıldırım et al. (2000). A 0.5 mL sample at varying concentrations was combined with 1.25 mL of 0.2 M phosphate buffer (pH 6.6) and 1.25 mL of 1% potassium ferricyanide solution. The mixtures were then incubated at 50°C for 30 min, followed by the addition of 1.25 mL of 10% (w/v) trichloroacetic acid. The resulting mixture was centrifuged at 3000×*g* for 10 min. Subsequently, 1.25 mL of the supernatant was mixed with 1.25 mL of distilled water and 0.25 mL of 0.1% (w/v) ferric chloride. Following a 10‐min reaction time, the absorbance of the solution was measured at 700 nm.

The β‐carotene bleaching test was evaluated according to the protocol of (Koleva et al., 2002). First, the emulsion of β‐carotene/linoleic acid was freshly prepared by dissolving 0.5 mg of β‐carotene (CAS 7235‐40‐7 | Sigma‐Aldrich), 25 μL of linoleic acid (CAS 60‐33‐3 | Sigma‐Aldrich), and 200 μL of Tween 40 in 1 mL of chloroform. After chloroform evaporation, 100 mL of distilled water was added, and the resulting mixture was vigorously stirred. In the test tube, 2 mL of the β‐carotene/linoleic acid emulsion were added to 0.5 mL of sample. In parallel, a control tube was prepared under the same conditions by adding 0.5 mL of water to the emulsion instead of sample. The absorbance was then measured at 470 nm before and after incubation for 1 h at 50°C. The antioxidant activity was evaluated in terms of β‐carotene bleaching by comparing the absorbance of each tube (test and control tubes) before and after the incubation period.

### 2.3 Animals treatments and experimental design

2.3.1

In this experimental study, male Wistar rats were used, each weighing between 170 and 180 g and aged about 10 weeks. Animals were obtained from the Central Society of Pharmaceutical Industries of Tunisia (SIPHAT, Ben‐Arous, Tunisia) and maintained under standard conditions (22 ± 0.5°C, 12/12 h light/dark cycle) with ad libitum access to food (standard pellet diet‐ Badr Utique‐TN) and water for a two‐week acclimatization period.

Weight and age‐matched animals were randomly assigned to eight groups, each consisting of six animals, and treated as follows:

- Corn oil (commercially available; brand Mazola), stripped of Vit E, was administered as a vehicle to the control group at a dosage of 0.4 mL/kg orally for 30 consecutive days
- The BPA (CAS 80‐05‐7 | Sigma‐Aldrich) group received an oral 100 mg/kg of BPA for 30 consecutive days (Grami et al., 2020).
- The EOMC group received an oral dose of 100 mg/kg of myrtle essential oil for 30 consecutive days.
- Myrtle 50 + BPA group received oral EOMC 50 mg/kg and BPA 100 mg/kg orally for 30 consecutive days
- Myrtle 100 + BPA group received oral EOMC 100 mg/kg and BPA 100 mg/kg orally for 30 consecutive days
- Myrtle 200 + BPA group received oral EOMC 200 mg/kg and BPA 100 mg/kg orally for 30 consecutive days
- Vit E (CAS 10191‐41‐0 | Sigma‐Aldrich) group received oral 100 mg/kg of vitamin E (α‐*tocopherol*) for 30 consecutive days (Oyeyemi et al., 2015)
- Vit E 100 + BPA group received oral vitamin E 100 mg/kg and BPA 100 mg/kg for 30 consecutive days.

To note, bisphenol A was introduced into groups IV, V, VI, and VIII after the 7th day of treatment.

The initial and final body weight of animals was measured, and the weight gain was calculated as the difference between final and initial values. However, food and water consumption were monitored daily.

### 2.4 Collection of specimens

After 30 days of treatment, all the rats were fasted for 12 h overnight and then sacrificed by decapitation. Blood was collected by intracardiac puncture, immediately centrifuged at 3000 rpm for 10 min at 4°C, and the separated plasma was kept at −80°C until use. The reproductive organs were removed immediately, rinsed with cold saline solution, and the absolute and relative weights were determined.

Sperm cells were obtained from the two‐cauda epididymis. The epididymis was removed and finely chopped in 1 mL of RPMI solution with 1000 IU/mL penicillin and 1000 μg/mL streptomycin to create a sperm suspension (Slimen & Gharbi 2014).

Total sperm count was assessed using specimens' samples from the right cauda epididymis, while sperm motility and morphology were analyzed using samples from the left cauda epididymis. Results were reported as the number of sperm per epididymis. To minimize potential errors, each sample was counted three times by three independent laboratory technicians trained and experienced. Ten microliters of sperm sample suspension were transferred onto a pre‐warmed microscope slide. Sperm motility was evaluated with a computer‐assisted sperm analysis system which categorized the sperm into progressive motile (actively moving), non‐progressive motile (slowly moving), and immotile (not moving) groups. The proportion of motile sperm in each observed field was calculated by dividing the number of motile sperm by the total sperm count, and the average across all fields was then determined. Sperm motility was presented as the percentage of motile sperm relative to the total sperm count.

The eosin staining technique was used to evaluate sperm viability. This method involved mixing one drop (10 μL) of freshly collected semen with two drops (20 μL) of saline‐eosin solution. Sperm cells with intact membranes and motility remained unstained, while non‐viable sperm cells showed purple to red staining. The dye exclusion test was performed on 100 spermatozoa, and sperm viability was determined as the percentage of dead sperm cells compared to the total cells (Grami et al., 2020).

Sperm morphology was assessed on a sperm suspension spread onto a slide, allowed to dry, and then fixed permanently. The slide was subsequently stained with 1% eosin following Seed's method involving the evaluation of sperm abnormalities by an optical microscope. A total of 100 spermatozoa from different fields on each slide were analyzed and categorized based on abnormalities observed in the head, tail, and tail‐head regions. The number of abnormal sperm cells was recorded, and the percentage was calculated (Grami et al., 2020).

### 2.5 Determination of sex hormone levels in plasma

Hormone levels in plasma were measured using commercially available ELISA kits (testosterone (CAS 58‐22‐0 | Sigma‐Aldrich), 17β‐estradiol (CAS 1743‐60‐8 | Sigma‐Aldrich), luteinizing hormone (CAS 39341‐83‐8 | Sigma‐Aldrich), follicle‐stimulating hormone (CAS 9002‐68‐0 | Sigma‐Aldrich)).

### 2.6 Biochemical evaluation of oxidative stress markers in reproductive organs

The reproductive organs were homogenized using a T‐18 digital ultra‐tracks homogenizer in TBS buffer (50 mM, pH 7.6). The homogenates were then centrifuged at 3000×*g* at 4°C for 15 min. The resulting supernatants were used to determine malondialdehyde (MDA) and protein levels, as well as the activity of antioxidant enzymes, including superoxide dismutase (SOD), catalase (CAT), and glutathione peroxidase (GPx), as well as glutathione (in terms of total thiol groups) as a non‐enzymatic antioxidant marker.

Protein concentrations were assessed using the method Bradford (1976), with bovine serum albumin serving as a reference standard.

The lipid peroxidation levels in the testes and epididymis were assessed by measuring malondialdehyde (MDA) concentration using the double heating method (Draper & Hadley, 1990). The molar extinction coefficient of the MDA‐TBA (CAS 84030‐12‐6 | Sigma‐Aldrich) complex is *ε* = 1.592 × 10^5^ M^−1^ cm^−1^. The results were expressed in nmol MDA/mg of protein.

The activity of superoxide dismutase (SOD) was determined using a modified epinephrine assay (Kakkar et al., 1984). In an alkaline environment, the superoxide anion facilitates the auto‐oxidation of epinephrine (CAS 51‐42‐3 | Sigma‐Aldrich) to adrenochrome. Changes in absorbance were monitored at 480 nm. Enzyme activity is reported in U/min/mg of protein.

Catalase (CAT) activity was assessed by measuring the initial rate of hydrogen peroxide disappearance at 240 nm (Aebi, 1974). The reaction mixture consisted of 33 mM H_2_O_2_ in 50 mM phosphate buffer at pH 7.0. The activity of CAT was determined using the extinction coefficient of 40 mM^−1^ cm^−1^ for H_2_O_2_.

The activity of glutathione peroxidase (GPx) was determined using the method Flohé & Günzler (1984).

The total thiol group concentration was performed on testis and epididymis homogenates upon mixing with 0.25 M Tris base and 20 mM ethylenediaminetetraacetic acid (EDTA) at pH 8.2 (Hu, 1994). The mixture was vortexed, and its absorbance was measured at 412 nm. The initial absorbance value was recorded as A1. Subsequently, 10 mM 5,5′‐dithiobis (2‐nitrobenzoic acid) (DTNB) was added. After an incubation time of 15 min, a new absorbance value, A2, was determined. The absorbance value of a white tube containing only DTNB and buffer was noted as B. The concentration of thiol groups (μmol/mg of protein) per tube was calculated using the formula: (A2 − A1 − B) × 1.57 mM.

### 2.7 Measurement of sex steroids (testosterone, progesterone, estradiol, and cortisol) in testicle by liquid chromatography–tandem mass spectrometry

Frozen tissue (whole testis) was transferred to 5 mL glass tubes and thawed. Buffer (0.5% BSA, w/v, 5 mM EDTA in PBS, pH 7.4) was added (500 mL per testis) before homogenization on ice for 20 s using an IKA T10 basic disperser set to maximum.

Homogenates were centrifuged (3000 rpm, 10 min, 4°C) to remove insoluble debris, and the supernatant was transferred to a new 1.5 mL tube and immediately prepared for LC–MS/MS analysis. For LC–MS/MS analysis, the tissue homogenate (200 mL), along with the standards (200 mL), were transferred into clean glass tubes and extracted with 1 mL of hexane: ethyl acetate (3:2 ratio) containing deuterated steroids as internal standards. The extracted samples were then allowed to phase separate at 4°C for 1 h before being placed in a freezer at −80°C for 30 min to freeze the lower aqueous layer. The upper organic layer containing the extracted target steroids was decanted into a clean glass tube and evaporated overnight at 37°C. The dried samples were reconstituted in 1.2 mL of 20% methanol in PBS. After thorough mixing, samples were transferred into 1.5 mL autosampler vials, and 1 mL was injected into a C8 column for analysis. Steroid concentrations were calculated as the amount per testis mass (McNamara et al., 2010).

### 2.8 Histology

Portions of the testis and epididymis were fixed in a 10% neutral buffered formalin solution, embedded in paraffin as previously described (Oliviero et al., 2021). Subsequently, 5 μm thick sections were cut, deparaffinized, hydrated, and stained with hematoxylin–eosin for analysis.

The area and diameter of the seminiferous tubules and the epididymis were measured by planimetry using ImageJ software.

### 2.9 Extraction and quantitative determination of lipids by LC–MS

For the LC‐QTOF analysis, a chromatograph Waters Acquity UPLC was used, coupled with a Waters Xevo G2 Quadrupole Time of Flight (QTOF) mass spectrometric (MS) detector. As the stationary phase, an Acquity Premiere BEH HILIC (2.1 × 100 mm, 1.7 μm) column was used, and the column temperature was maintained at 40°C. Mixtures of water with 0.1% formic acid (A) and acetonitrile with 0.1% formic acid (B) were used as the mobile phases. The elution gradient was as follows: 0–1 min, 10% A; 15 min, 25% A; 18 min, 30% A. The flow rate used was 0.2 mL/min and the injection volume was 1 μL. MS data were acquired in positive ionization mode (ESI+) in the mass range 50–2000 Da. The sampling cone voltage was adjusted to 40 V, and the source offset to 80 V. The capillary voltage was adjusted to 3.5 kV. The nebulizer gas used was N_2_ at a flow rate of 800 L/h. The desolvation temperature was 450°C. The mass accuracy and reproducibility were maintained by infusing lock mass through Lockspray at a flow rate of 20 μL/min. The *m*/*z* value of all acquired spectra was automatically corrected during acquisition based on lock mass. A MSe experiment was simultaneously performed to collect structural information, setting the collision energy to 30 V. Compounds were identified on the basis of HR‐MS and MSe fragmentation.

### 2.10 Statistical analysis

All results were presented as mean ± SD. Statistical analysis was performed using one‐way analysis of variance (ANOVA) with GraphPad Prism statistical software, Version 9.0.2 (GraphPad Software Inc., La Jolla, CA, USA), to compare between groups. When significant differences were found, post hoc comparisons were performed using Tukey's HSD to correct for multiple testing. *p*‐values < 0.05 were considered statistically significant.

## 3 RESULTS

### 3.1 In vitro antioxidant activity of EOMC

**FIGURE 1:**
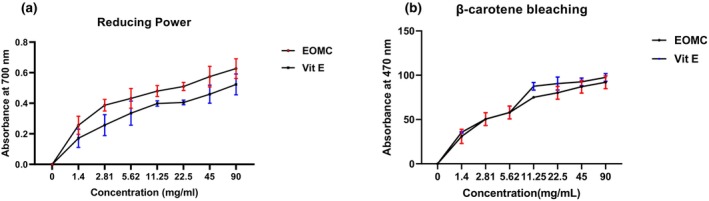
Dose response of the antioxidant capacity of EOMC or Vit E in the Reducing Power test (a) and β‐carotene bleaching test (b), (mean ± SD [*n* = 3]).

The antioxidant activity of EOMC by the evaluation of reducing power and β‐carotene bleaching tests are shown in Figure 1.

The reducing capacity of EOMC and the standard Vit E increased in a concentration‐dependent manner (Figure 1a). The maximum value (0.6 ± 0.06) was achieved by EOMC, which was significantly greater than that observed for Vit E (0.5 ± 0.07), indicating that EOMC had higher antioxidant capacity than Vit E.

### 3.2 β‐carotene bleaching test

Both EOMC and Vit E showed effective inhibition of linoleic acid‐β‐carotene oxidation (Figure 1b). The IC_50_ value for the EOMC was 1.98 mg/mL ±0.76, while the IC_50_ value for Vitamin E was about 0.61 mg/mL ±0.21.

### 3.3 Effects on rat weight gain, food and water intake, and weight of reproductive organs

**FIGURE 2:**
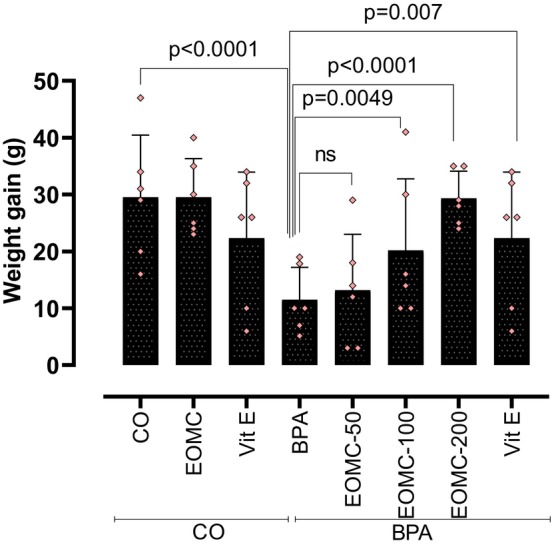
Effect of EOMC on the Weight Gain After Subchronic Treatment with BPA at 100 mg/kg for 30 days. Number of rats: *n* = 6/group. BPA, bisphenol A; CO, corn oil control group; EOMC, essential oil of *myrtus comminus*; Vit E, vitamin E; EOMC‐BPA, essential oil of *myrtus comminus* at (50, 100, and 200 mg/kg) with bisphenol A; Vit E‐BPA, Vitamin E with bisphenol A. Values are the mean ± SD; *p* values of the compared conditions are indicated.

The effect of the different exposure conditions on the absolute weight gain of animals are reported in Figure 2 and the corresponding mean overall food and water intake is reported in Table 1. In control conditions, administration of EOMC had no significant effect compared to animals undergoing sham treatment (*p* > 0.05). Interestingly, administration of Vit E was associated with a significant reduction of the weight gain with no apparent involvement of food intake. As expected, exposure to BPA was associated with a much‐reduced weight gain compared to sham control and a corresponding reduction of both food and water intake (*p* < 0.05 vs. CO). The co‐administration of EOMC with BPA was associated with a significant and dose‐dependent recovery of both weight gain and food/water intake. However, the effects of the co‐administration of Vit E significantly recovered the weight gain less pronounced than the weight gain observed after co‐administration of EOMC with BPA. Thus, under the conditions of the experiment, EOMC exerted a stronger protective effect on body weight and feeding behavior than Vit E.

**TABLE 1:** Effect of essential oil of *myrtus comminus* on water and food intake after the subchronic treatment with bisphenol A at 100 mg/kg for 30 Day.

	Daily food intake	Daily water intake
Control	136.33 ± 11.40	201.56 ± 14.09
BPA	105.32 ± 26.38^a^	105.56 ± 16.37^a^
EOMC	143.87 ± 9.05^b^	262 ± 13.30^a^, ^b^
EOMC‐50 + BPA	122.13 ± 19.20^b^	125 ± 13.38^a^, ^b^
EOMC‐100 + BPA	126.57 ± 25.09^b^	170.23 ± 23.08^a^, ^b^
EOMC‐200 + BPA	136.92 ± 15.51^b^	186.33 ± 24.42^b^
Vit E + BPA	112.9 ± 21.43^a^	154 ± 26.04^a^, ^b^
Vit E	122.17 ± 16.61^b^	173.33 ± 30.03^a^, ^b^

*Note*: Number of rats: *n* = 6/group. Values are the mean ± SD.

Abbreviations: BPA, bisphenol A; Control, negative control group; EOMC‐100, essential oil of *myrtus comminus*; EOMC‐BPA, essential oil of *myrtus comminus* at (50,100, and 200 mg/kg) with bisphenol A; Vit E, Vitamin E; Vit E‐BPA, Vitamin E with bisphenol A.

^a^ *p* <0.05 compared to control group.

^b^ *p* <0.05 compared to BPA group (ANOVA test).

The effect of the different exposure conditions on the testis and epididymal weights of animals are reported in Table 2. Compared to sham control, exposure to BPA was associated with a significant reduction of both testis and epididymal weights, whether absolute or relative to body weight. The co‐administration of three different doses of EOMC was associated with a significant restoration of organs' weight even with the lowest dosage used. Similar effects were recorded for Vit E co‐administration (Table 2).

**TABLE 2:** Effect of essential oil of *myrtus communis* on the weight of reproductive organs after the subchronic treatment with bisphenol A at 100 mg/kg b.w. for 30 Days.

	Testicles		Epididymis	
	Absolute weight (g)	Relative weight	Absolute weight (g)	Relative weights
Control	2.29 ± 0.19	0.79 ± 0.08	1.081 ± 0.45	0.37 ± 0.16
BPA	1.068 ± 0.10^a^	0.41 ± 0.04^a^	0.73 ± 0.11^a^	0.28 ± 0.04^a^
EOMC‐100	2.69 ± 0.16^a^, ^b^	0.96 ± 0.07^a^, ^b^	1.55 ± 0.26^a^, ^b^	0.55 ± 0.09^a^, ^b^
EOMC‐50 + BPA	2.14 ± 0.60^b^	0.81 ± 0.22^b^	1.038 ± 0.42^b^	0.39 ± 0.15^b^
EOMC‐100 + BPA	2.31 ± 0.34^b^	0.83 ± 0.10^b^	1.11 ± 0.36^b^	0.40 ± 0.12^b^
EOMC‐200 + BPA	2.6 ± 0.26^a^, ^b^	0.93 ± 0.10^a^, ^b^	1.41 ± 0.16^a^, ^b^	0.50 ± 0.05^a^, ^b^
Vit E + BPA	2.27 ± 0.35^b^	0.82 ± 0.13^b^	0.97 ± 0.27	0.35 ± 0.10
Vit E	2.31 ± 0.35^b^	0.83 ± 0.11^b^	0.99 ± 0.35^b^	0.36 ± 0.13

*Note*: Number of rats: *n* = 6/group. Values represents the paired weights of both testes and both epididymis. Values are the mean ± SD.

Abbreviations: BPA, bisphenol A; Control, negative control group; EOMC‐100, essential oil of *myrtus comminus*; EOMC‐BPA, essential oil of *myrtus comminus* at (50,100, and 200 mg/kg) with bisphenol A; Vit E, Vitamin E; Vit E‐BPA, Vitamin E with bisphenol A.

^a^ *p* <0.05 compared to control group.

^b^ *p* <0.05 compared to BPA group (ANOVA test).

### 3.4 Effect on semen parameters and circulating sex hormones

The effect of the different exposure conditions on semen parameters is reported in Tables 3 and 4. Compared to sham control animals, exposure to BPA was associated with a significant reduction in total sperm count, cell motility, and viability. In addition, a significant increase in the percentage of sperms with morphological abnormalities, particularly at the head and tail levels, was observed. However, the co‐administration of increasing doses of EOMC was associated with a significant recovery of sperm parameters from the lowest dosage used (50 mg/kg). The co‐administration of Vit E was also associated with a significant improvement in sperm parameters.

**TABLE 3:** Effects of essential oil from *Myrtus communis* on spermatozoa on performance quality.

	Total sperm count (10^6^ cells)	Motile spermatozoa (%)	Viabile spermatozoa (%)
Control	60.8 ± 0.26	85.5 ± 5.63	86 ± 0.34
BPA	30.23 ± 0.24^a^	22 ± 1.22^a^	55 ± 0.24^a^
EOMC‐100	60.94 ± 0.48^b^	87.69 ± 2.49^b^	87.90 ± 1.22^a^, ^b^
EOMC‐50 + BPA	50.12 ± 0.58^a^, ^b^	54.48 ± 5.63^a^, ^b^	59 ± 0.53^a^, ^b^
EOMC‐100 + BPA	60.01 ± 0.78^a^, ^b^	65.14 ± 10.04^a^, ^b^	68 ± 0.44^a^, ^b^
EOMC‐200 + BPA	60.7 ± 0.61^b^	77.29 ± 1.46^a^, ^b^	80 ± 0.56^a^, ^b^
Vit E + BPA	60 ± 0.34^a^, ^b^	68.05 ± 3.42^a^, ^b^	77 ± 0.78^a^, ^b^
Vit E	60.5 ± 0.44^b^	79 ± 4.89^ab^	82 ± 0.56^a^, ^b^

*Note*: Number of rats: *n* = 6/group. Values are the mean ± SD.

Abbreviations: BPA, Bisphenol A; Control, negative control group; EOMC‐100, essential oil of *myrtus comminus*; EOMC‐BPA, essential oil of *myrtus comminus* at (50,100, and 200 mg/kg) with bisphenol A; Vit E, Vitamin E; Vit E‐BPA, Vitamin E with bisphenol A.

^a^ *p* <0.05 compared to control group.

^b^ *p* <0.05 compared to BPA group (ANOVA test).

**TABLE 4:** Effects of essential oil from *Myrtus communis* on sperm morphology.

	Morphology		
	Normal form	Head abnormalities	Tail abnormalities
Control	80 ± 0.12	15 ± 2.44	5 ± 0.12
BPA	10 ± 0,61^a^	20 ± 5.87^a^	70 ± 0.80^a^
EOMC‐100	85.5 ± 0.26^a^, ^b^	8.6 ± 0.12^a^, ^b^	5.9 ± 0.61^a^, ^b^
EOMC‐50 + BPA	51 ± 0.48^a^, ^b^	13.4 ± 5.38^b^	55.6 ± 0.44^a^, ^b^
EOMC‐100 + BPA	65.7 ± 0.48^a^, ^b^	12.3 ± 0.29^a^, ^b^	22 ± 0.58^a^, ^b^
EOMC‐200 + BPA	70 ± 0.78^a^, ^b^	11 ± 0.56^a^, ^b^	19 ± 0.78^a^, ^b^
Vit E + BPA	85 ± 0.29^a^, ^b^	10 ± 2.93^a^, ^b^	5 ± 0.56^b^
Vit E	92.3 ± 0.29^a^, ^b^	4.5 ± 0.12^a^, ^b^	3.2 ± 0.44^a^, ^b^

*Note*: Number of rats: *n* = 6/group. Values are the mean ± SD.

Abbreviations: BPA, bisphenol A; Control, negative control group; EOMC‐100, essential oil of myrtus comminus; EOMC‐BPA, essential oil of myrtus comminus at (50,100, and 200 mg/kg) with bisphenol A; Vit E, Vitamin E; Vit E‐BPA, Vitamin E with bisphenol A.

^a^ *p* <0.05 compared to control group.

^b^ *p* <0.05 compared to BPA group (ANOVA test).

**FIGURE 3:**
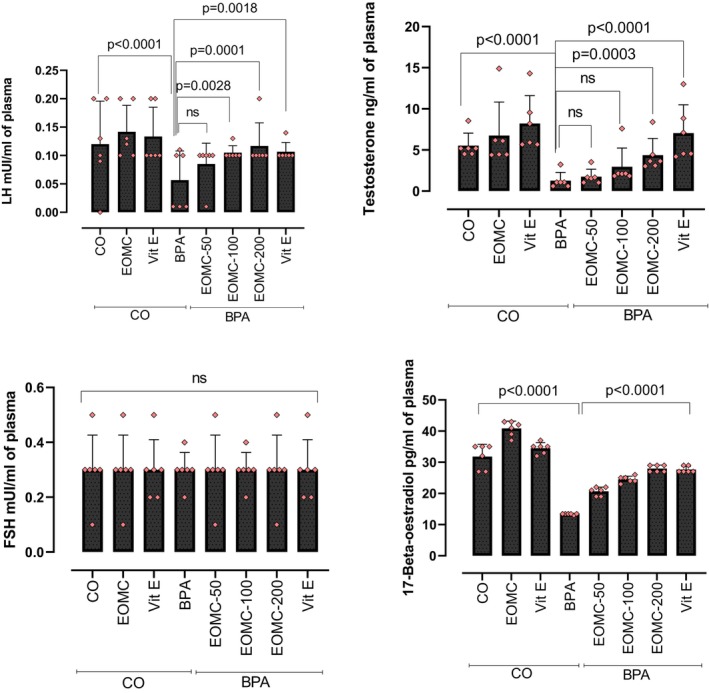
Effect of EOMC on reproductive hormones in plasma after subchronic treatment with BPA at 100 mg/kg. Number of rats: *n* = 6/group. BPA, bisphenol A; CO, corn oil control group; EOMC, essential oil of *myrtus communis*; EOMC‐BPA, essential oil of *Myrtus communis* at (50, 100, and 200 mg/kg) with bisphenol A; Vit E, Vitamin E; Vit E‐BPA, Vitamin E with bisphenol A. Values are the mean ± SD; *p* values of the compared conditions are indicated.

The effect of the different conditions on plasma levels of luteinizing hormone (LH), follicle stimulation hormone (FSH), testosterone, and *17β*‐*estradiol*(E2) is shown in Figure 3. Compared to sham control, BPA exposure was associated with a great reduction of testosterone, E2, and LH levels. However, the co‐administration of EOMC with BPA was associated with a significant and dose‐dependent improvement in all hormones. For both testosterone and LH, a significant increase in serum levels was observed at an EOMC dose equal to or greater than 100 mg/kg. Nevertheless, no significant effects were observed on FSH levels in any group. In addition, EOMC displayed a more pronounced improvement in plasma 17β‐estradiol compared to Vit E, whereas no marked differences were observed between the two treatments for effects on testosterone, LH, and FSH.

### 3.5 Effect on testes and epididymis redox state

**FIGURE 4:**
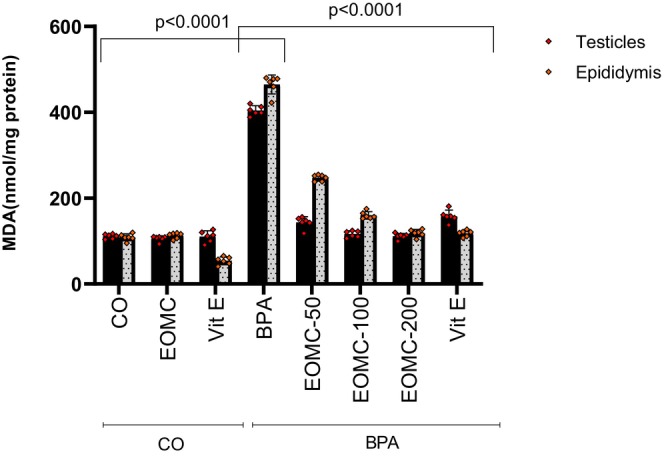
Effect of EOMC on the MDA in testes and epididymis after subchronic treatment with BPA at 100 mg/kg. Number of rats: *n* = 6/group. BPA, bisphenol A; CO, corn oil control group; EOMC, essential oil of *Myrtus communis*; EOMC‐BPA, essential oil of *Myrtus communis* at (50, 100 and 200 mg/kg) with bisphenol A; Vit E, Vitamin E; Vit E‐BPA, Vitamin E with bisphenol A. Values are the mean ± SD; *p* values of the compared conditions are indicated.

The effect of the different exposure conditions on testis and epididymis lipid peroxidation, evaluated by the MDA assay, is reported in Figure 4. The Vit E treatment resulted in a reduction of lipid peroxidation in control epididymis, compared to sham control. As expected, the exposure to BPA was associated with a significant increase in the MDA level for both testis and epididymis tissues, compared to sham control animals. The co‐administration of EOMC was associated with a large and significant reduction in MDA concentration for both tissues from the lowest used dose (50 mg/kg). When comparing the protective effects, EOMC exerted a stronger reduction in lipid peroxidation in the testes, while vitamin E displayed a more pronounced effect in the epididymis.

**FIGURE 5:**
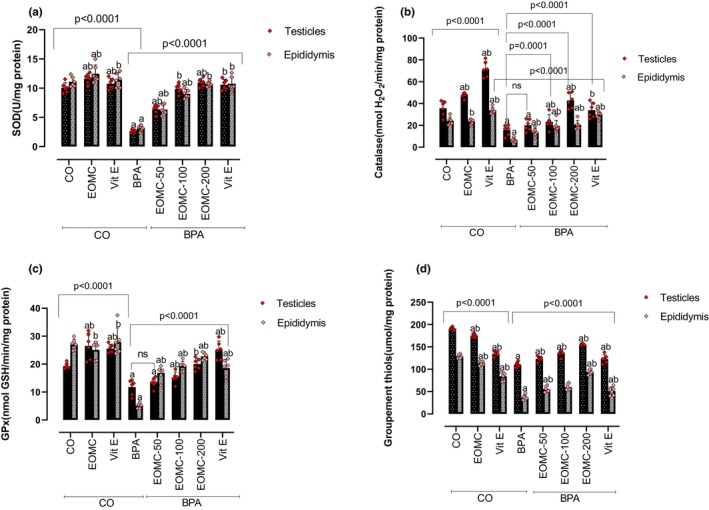
Effect of EOMC on the SOD (a), CAT (b), GPx activities (c), and Groupement thiols (d) in testes and epididymis after subchronic treatment with BPA at 100 mg/kg. Number of rats: *n* = 6/group. BPA, bisphenol A; CO, corn oil control group; EOMC, essential oil of *myrtus comminus*; EOMC‐BPA, essential oil of *myrtus comminus* at (50, 100 and 200 mg/kg) with bisphenol A; Vit E, Vitamin E; Vit E‐BPA, Vitamin E with bisphenol A. Values are the mean ± SD; *p* values of the compared conditions are indicated.

The effect of EOMC or Vit E on SOD, CAT, GPx activities and total thiol content was also investigated (Figure 5). Compared to sham controls, SOD activity was strongly and significantly blunted upon BPA exposure. A dose‐dependent recovery was observed when EOMC was co‐administrated, with no differential pattern between testis and epididymis (Figure 5a). Enzyme activities were almost restored to control levels. Similar effects were observed for the CAT and GPx activities (Figure 5b,c). On the other hand, for the total thiol content in both tissues (testis and epididymis), a significant reduction was observed upon BPA exposure, compared to sham controls, but it was significantly restored by EOMC at 200 mg/kg (Figure 5d). EOMC co‐administration with BPA exerted a more pronounced improvement of SOD activity and thiol content in both testis and epididymis, while Vit E co‐administration resulted in a relatively stronger effect on CAT and GPx activities.

### 3.6 Effect on sex steroids in the testis

The effect of different exposure conditions on sex steroids, including testosterone, estradiol, progesterone, and cortisol, is shown in Table 7. Compared to sham control animals, BPA exposure was associated with a reduction in testosterone, estradiol, progesterone, and cortisol. However, co‐administration of increasing doses of EOMC was associated with a significant improvement in all steroid hormones. By comparison, Vit E induced a significantly greater improvement in testosterone and cortisol levels than EOMC, while no marked difference was observed between the two treatments in effects on estradiol and progesterone.

### 3.7 Metabolomic profiling of lipid composition in sperm cells: Effects of BPA and protective treatments with EOMC and Vit E

**FIGURE 6:**
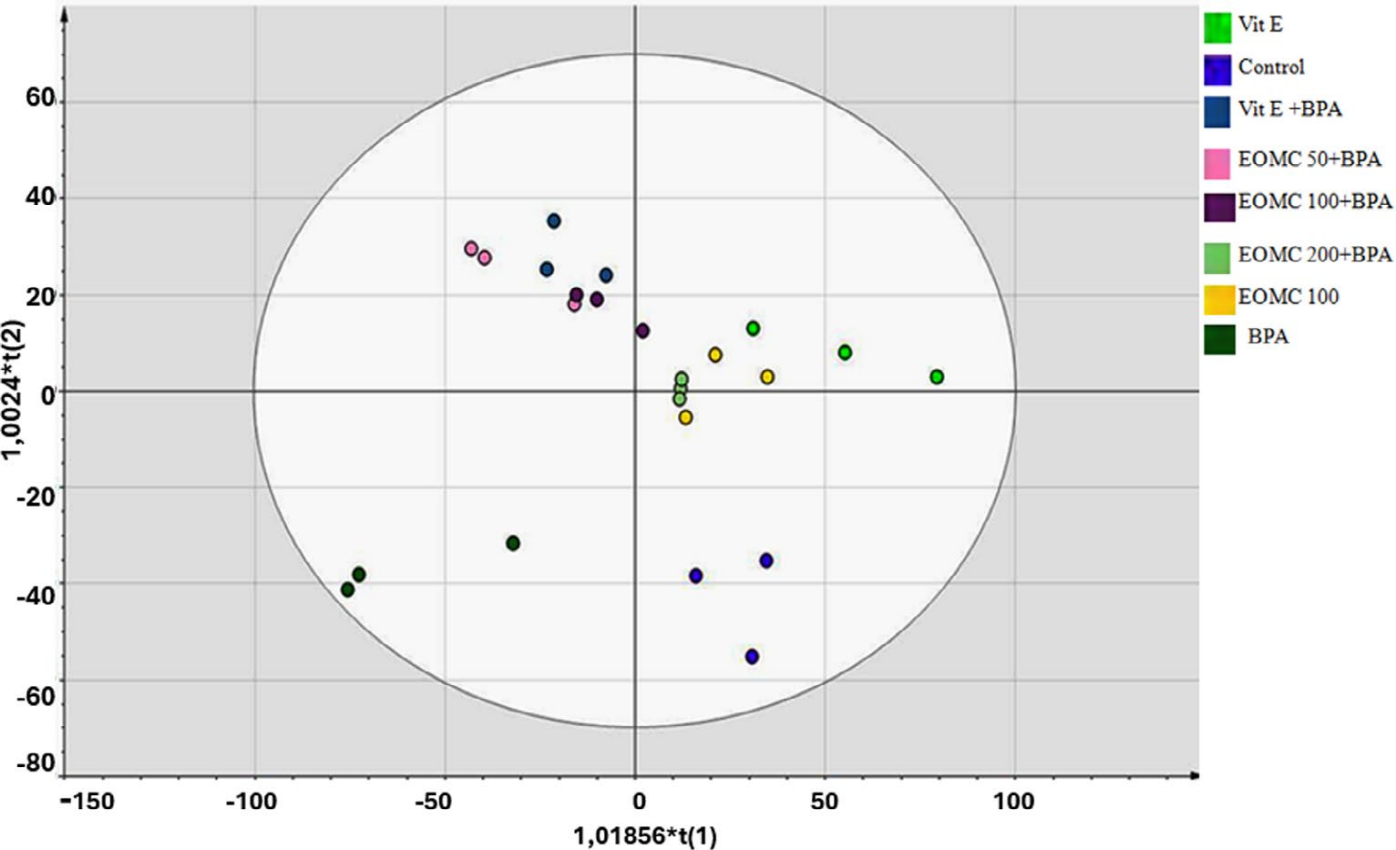
OPLS‐DA score plot illustrating the effects of BPA, EOMC, and Vit E on sperm lipid composition in rats. BPA, bisphenol A (100 mg/kg); Control, negative control (corn oil); EOMC + BPA, *Myrtus communis* essential oil (50, 100, and 200 mg/kg) with BPA; EOMC, essential oil of *Myrtus communis*; Vit E + BPA, Vitamin E with BPA; Vit E, Vitamin E.

The lipid fraction of sperm cells from the different animal groups was extracted to evaluate the possible changes in composition, caused by the different exposure conditions (Table 6, Figure 6). In order to establish the possible variations in phospholipid composition, an untargeted metabolomic approach was used. All the MS data were extracted using the software MZmine and a matrix combining the acquired MS spectra and respective retention times was built. The elaboration was then performed using Simca 12 and different multivariate models were obtained (Figure 6). At first, the PCA analysis showed a differential clustering between control animals versus the BPA‐treated groups. The graphic representation of the OPLS‐DA model showed a differential and homogeneous clustering of all replicates of control conditions: sham, BPA, vitamin E, and EOMC (Figure 6). In qualitative terms, co‐administration of EOMC showed a progressive and dose‐dependent overlap to control EOMC conditions. A similar trend was observed in animals receiving Vit E. In quantitative terms, membrane lipids associated with the highest variance among groups are reported in Table 6. Interestingly, exposure to BPA was associated with massive enrichment in phosphatidylcholine and significant increase of saturated diacyl‐glycerol DG (PGF1alpha/0:0/i‐22:0), whereas a significant reduction of semenolipid was observed. Co‐administration of EOMC was not effective in restoring semenolipid content or lowering phosphatidylcholine, but it was associated with an increase of glycosphingolipids and lysophosphatidic acid.

### 3.8 Testis histology and morphometry

**FIGURE 7:**
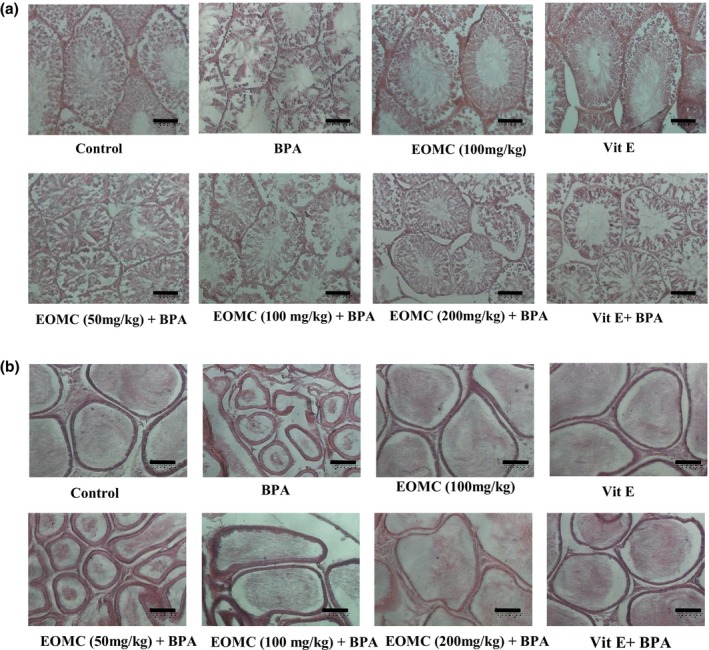
(a) Histopathological changes in rat testes following subchronic exposure to BPA and protective treatment with EOMC. (Scale bar, 20 μm). BPA, bisphenol A (100 mg/kg); (b) Histopathological changes on the cauda epididymis of rat following subchronic exposure to BPA and protective treatment with EOMC. (Scale bar, 20 μm). BPA, bisphenol A (100 mg/kg); Control, negative control (corn oil); EOMC + BPA, *Myrtus communis* essential oil (50, 100, and 200 mg/kg) with BPA; EOMC, essential oil of *Myrtus communis*; Vit E + BPA, Vitamin E with BPA; Vit E, Vitamin E.

Histological analysis of testicular tissue samples stained with hematoxylin–eosin in the control group (Figure 7) revealed a regular and normal number of seminiferous tubules, separated by connective tissue and interstitial cells. Similar observations were made in the seminiferous tubules of rats treated with EOMC in combination with 100 mg/kg of BPA. However, in the group treated exclusively with 100 mg/kg of BPA, the hematoxylin–eosin‐stained testicular sections showed numerous histological alterations, including disorganization of the seminiferous epithelium, vacuolization, sloughing of germ cells into the lumen, and a reduction in the number of mature spermatozoa compared to the control group.

Regarding testicular and epididymal morphometry (Table 5), a significant reduction in the area and diameter of the seminiferous and epididymal tubules was observed in the testes of BPA‐treated animals. However, co‐treatment with EOMC or Vit E protected against these alterations.

**TABLE 5:** Effect of essential oil of *Myrtus communis* on morphometry of testes and epididymis.

Groups	Area of seminiferous tubule (%)	Diameter of the seminiferous tubule (μm)	Area of epididymal (%)	Diameter (μm)
Control	69.67 ± 10.77	244.80 ± 11.88	63.94 ± 4.88	395.79 ± 8.59
BPA	35.13 ± 7.08^a^	196.44 ± 11.62^a^	41.47 ± 9.01^a^	225.99 ± 7.33^a^
EOMC‐100	71.35 ± 5.90^b^	249.61 ± 11.37^b^	66.49 ± 9.23^b^	482.55 ± 9.81^a^, ^b^
EOMC‐50 + BPA	43.12 ± 8.84^a^	203.74 ± 10.68^a^	44.62 ± 9.47^a^	390.95 ± 11.95^b^
EOMC‐100 + BPA	51.99 ± 5.93^a^, ^b^	239.12 ± 10.51^b^	56.78 ± 7.96^b^	399.57 ± 9.11^b^
EOMC‐200 + BPA	64.76 ± 9.44^b^	247.49 ± 7.16^b^	60.41 ± 5.85^b^	431.07 ± 6.47^a^, ^b^
Vit E + BPA	62.46 ± 9.65^b^	248.9 ± 9.49^b^	63.03 ± 3.40^b^	426.96 ± 8.20^a^, ^b^
Vit E	69.97 ± 9.56^b^	253.33 ± 12.93^b^	66.17 ± 7.05^b^	444.06 ± 7.69^a^, ^b^

^a^ *p* <0.05 compared to control group.

^b^ *p* <0.05 compared to BPA group (ANOVA test).

**TABLE 6:** Effects of bisphenol A, myrtle essential oil, and Vitamin E on sperm lipid composition in rats: concentrations of key phospholipids and glycosphingolipids.

	Control	BPA	EOMC	EOMC‐50 + BPA	EOMC‐100 + BPA	EOMC‐200 + BPA	Vit E + BPA	Vit E
Seminolipid	105.44 ± 5.35	62.70 ± 7.53^a^	99.40 ± 1.78^b^	65.06 ± 15.62^a^	73.58 ± 6.36^a^	85.72 ± 5.73^a^, ^b^	68.47 ± 24.39^a^	78.94 ± 6.35^a^
Cardiolipin (20:5 18:2)	140.35 ± 21.66	129.46 ± 3.16	164.26 ± 13.94^b^	138.056 ± 12.44	142.67 ± 4.22	163.56 ± 3.26^b^	139.63 ± 34.82	167.34 ± 7.33^a^, ^b^
Phosphatidylcholine	0 ± 0	188.78 ± 64.84^a^	76.58 ± 0^b^	0 ± 0	0 ± 0	0 ± 0	0 ± 0	140.96 ± 22.04^a^, ^b^
Cardiolipin (20:4 18:1)	0 ± 0	0 ± 0	22,65 ± 19.76^ab^	0 ± 0	0 ± 0	0 ± 0	0 ± 0	62.04 ± 20.69^a^, ^b^
Glycosphingolipids	68.50 ± 7.17	43.87 ± 11.58	83.83 ± 2.93^b^	48.88 ± 19.76	75.88 ± 10.51^b^	89.85 ± 3.49^b^	60.07 ± 10.16	103.44 ± 31.79^a^, ^b^
DG (PGJ2/0:0/i‐20:0)	749.90 ± 84.24	286.38 ± 51.68^a^	662.39 ± 60.71^b^	338.78 ± 18.66^a^	454.64 ± 70.20^a^, ^b^	537.46 ± 21.95^a^, ^b^	384.43 ± 67.80^a^	835.19 ± 95.70^b^
Cardiolipin	15.23 ± 13.40	4.22 ± 7.31^a^	14.23 ± 2.28	5.20 ± 9	11.66 ± 10.35	12.63 ± 1.13	4.3 ± 4.37^a^	11.55 ± 10
Phosphatidylethanolamine (O‐18:0/18:2)	169.49 ± 27.44	197.46 ± 72.17	94.97 ± 5.52^a^, ^b^	137.69 ± 48.65^b^	100.50 ± 5.84^a^, ^b^	83.95 ± 3.51^a^, ^b^	97.57 ± 1.85^a^, ^b^	87.84 ± 3.94^a^, ^b^
DG (PGF1alpha/0:0/i‐22:0)	120.20 ± 15.84	157.32 ± 11.77^a^	78.68 ± 2.76^a^, ^b^	98.44 ± 3.38^ab^	87.62 ± 5.50^a^, ^b^	80.54 ± 13.67^a^, ^b^	103.32 ± 10.60^a^, ^b^	84.55 ± 2.37^a^, ^b^
Oxidized phosphatidylserine	0 ± 0	0 ± 0	4.16 ± 7.21^a^, ^b^	0 ± 0	0 ± 0	0 ± 0	0 ± 0	15.26 ± 0^a^, ^b^
LysoPA	3.92 ± 6.79	0 ± 0	13.62 ± 1.56^a^, ^b^	5.39 ± 9.34	12.09 ± 10.58^a^, ^b^	15.23 ± 0.12^a^, ^b^	4.61 ± 4.61	21.11 ± 2.05^a^, ^b^

Abbreviations: BPA, bisphenol A (100 mg/kg); Control, negative control (corn oil); EOMC + BPA: *Myrtus communis* essential oil (50, 100, and 200 mg/kg) with BPA; EOMC, essential oil of *Myrtus communis*; Vit E + BPA, Vitamin E with BPA; Vit E, Vitamin E.

^a^ *p* <0.05 compared to control group.

^b^ *p* <0.05 compared to BPA group (ANOVA test).

**TABLE 7:** Effects of bisphenol A, myrtle essential oil, and Vitamin E on sex steroids in testes in rat.

Groupes	Testosterone (pg/g)	Estradiol (pg/g)	Progesterone (pg/g)	Cortisol (pg/g)
Control	1.83 ± 1.51	1.11 ± 1.12	0.077 ± 0.05	0.30 ± 0.19
BPA	0.66 ± 1	0.09 ± 0.02^a^	0.025 ± 0.01^a^	0.20 ± 0.12
EOMC‐100	2.94 ± 4.38^b^	2.40 ± 0.85^a^, ^b^	0.17 ± 0.04^a^, ^b^	1.50 ± 1.51^a^, ^b^
EOMC‐50+ BPA	1.39 ± 0.95	0.11 ± 0.02^a^	0.039 ± 0.01	0.65 ± 0.22
EOMC‐100+ BPA	2.12 ± 0.97^b^	0.36 ± 0.19^a^	0.075 ± 0.04^b^	0.83 ± 0.46^a^, ^b^
EOMC‐200+ BPA	2.83 ± 3.18^b^	1.60 ± 1.46^b^	0.11 ± 0.14^b^	1.23 ± 0.51^a^, ^b^
Vit E + BPA	0.70 ± 0.29	0.65 ± 0.36	0.033 ± 0.009	0.24 ± 0.12
Vit E‐100	4.95 ± 2^a^, ^b^	2.07 ± 1.51^a^, ^b^	0.17 ± 0.073^a^, ^b^	0.58 ± 0.41

*Note*: Number of rats: *n* = 6/group. Values are the mean ± SD.

Abbreviations: BPA, bisphenol A; Control, negative control group; EOMC‐100, essential oil of *myrtus comminus*; EOMC‐BPA, essential oil of *Myrtus comminus* at (50,100, and 200 mg/kg) with bisphenol A; Vit E, Vitamin E; vit E‐BPA, Vitamin E with bisphenol A.

^a^ *p* <0.05 compared to control group.

^b^ *p* <0.05 compared to BPA group (ANOVA test).

## 4 DISCUSSION

The objective of the present study was to assess the pharmacological effects of EOMC on reproductive toxicities and damage induced by endocrine disruptors in adult male rats exposed to BPA. Initial experiments, involving the assessment of free radicals using two methods (reducing power and β‐carotene bleaching), demonstrated that EOMC exhibited a strong capacity to scavenge free radicals and to reduce ions. This antioxidative effect is due to key compounds such as 1,8‐cineole and α‐pinene, which act synergistically in the studied oil (Cherrat et al., 2014). Given this robust antioxidant activity, the essential oil has the potential to neutralize reactive oxygen species (ROS), thus preventing damage to proteins, lipids, and DNA, which are major factors in cellular aging caused by oxidative stress (Ibrahim et al., 2021). In fact, α‐pinene and 1,8‐cineole (major compounds in EOMC) activate the transcription factor Nrf2, increasing the expression of CAT, SOD, GPx, GR, HO‐1, and other antioxidant enzymes (Porres‐Martínez et al., 2016).

For the in *vivo* analysis, BPA was administered orally at a toxic dose of 100 mg/kg body weight per day. Previous research has shown that this level of exposure disrupted the reproductive system and exceeded no‐observed‐adverse‐effects, as reported by the Karnam study, showing reproductive toxicity at doses of 50 mg/kg (Karnam et al., 2015). The current data corroborate that male rats exposed to BPA (100 mg/kg) had reproductive toxicity and testicular damage (Grami et al., 2020; Kamel et al., 2018). Furthermore, the present study observed a decrease in water and food intake, which is consistent with the findings of Grami et al., where BPA administration led to reduced water/food intake, suggesting a possible interaction with appetite and thirst regulation mechanisms (Nuñez et al., 2021).

The study also revealed a reduction in body weight, which is an important marker of reproductive toxicity in male animals. These findings align with Gurmeet and colleagues' research, which demonstrated weight loss in rats exposed to BPA at doses of 1, 5, and 100 mg/kg (Miao et al., 2008). However, Gules et al. (Gules et al., 2019) did not report a significant weight loss at a dose of 50 mg BPA/kg. It is important to recognize the variability in BPA dosing and exposure duration across studies, which may influence the results. Additionally, weight loss could be attributed to metabolic disruptions, as BPA interferes with glucose and lipid metabolism (Miao et al., 2008). Jin et al. (Jin & Zhou, 2020) also reported a significant decrease in testicular weight following exposure to 100 mg/kg of BPA.

The impact of BPA on male reproductive organs remains controversial. For instance, Ema et al. (Ema et al., 2001) found no significant changes in reproductive parameters in rats treated with BPA at doses ranging from 0.2 to 200 mg/kg over two generations. Weight loss could also be related to reduced testosterone bioavailability, as blood testosterone levels are indicative of reproductive function (Selmi et al., 2018). Additionally, a decrease in 17β‐estradiol levels was observed in the BPA‐exposed group, which is consistent with BPA's role as an endocrine disruptor able to interact with estrogen (ER) and androgen (AR) receptors (Tonini et al., 2020). While no variation in FSH levels was observed in this study, Akintunde et al. (2019) reported that a BPA dose of 50 mg/kg BW significantly reduced FSH levels (Akintunde et al. 2019). In addition, the effect of BPA on sex steroids remains poorly studied, and assay methods vary from study to study. In our work, we used liquid chromatography‐mass spectrometry (LC–MS) and demonstrated that BPA induces a decrease in steroid hormones such as testosterone, estradiol, progesterone, and cortisol in the testicle. These results are consistent with those reported by Nakamura et al. (2010) who showed, using RIA, radioimmunoassay, that administration of BPA at 100 mg/kg resulted in a decrease in testicular testosterone levels (Nakamura et al., 2010). However, co‐administration of EOMC at different concentrations restored the levels of the analyzed steroid hormones. To our knowledge, no previous study has explored the effects of EOMC in this context. In contrast, the study by Azza M. El‐wakf et al. 2015 showed that marjoram (*Origanum majorana*) and sage (*Salvia officinalis*) essential oils enhance the activity of key enzymes in steroidogenesis, such as 3β‐HSD, CYP17A1, and CYP19A1 (aromatase) (El‐Wakf et al., 2015), which are involved in the conversion of cholesterol into active hormones (Payne & Hales, 2004).

Importantly, sperm parameter analysis in this study revealed that BPA treatment significantly reduced sperm motility, viability, morphology, and testicular histological alterations including disorganization of the seminiferous epithelium, vacuolization, sloughing of germ cells into the lumen, and a reduction in the number of mature spermatozoa compared to the control group. However, co‐treatment with increasing concentrations of EOMC demonstrated a protective effect, and even in the absence of BPA, the essential oil improved sperm quality. This is aligned with the findings of Mbaye et al., who showed that essential oils from sage (*Salvia officinalis*), oregano (*Origanum vulgare*), and eucalyptus (*Eucalyptus globulus*) positively affected human sperm parameters (Mbaye et al., 2019). Moreover, Oliveira Santos et al. highlighted the antioxidant properties of *Syzygium aromaticum* essential oil in bovine sperm (Santos et al., 2019).

BPA, a well‐established endocrine disruptor, poses a significant threat to sperm cell membranes due to their lipid‐rich composition, particularly in phospholipids. The phospholipid structure of these membranes is highly susceptible to damage from toxic agents. In this study, the LC/MS/MS tool was used to analyze phospholipid alterations induced by BPA exposure and to evaluate the protective effects of EOMC and vit E, which served as a reference antioxidant compound.

Given their pivotal role in spermatogenesis and sperm‐egg interaction (Kongmanas et al., 2021), the analysis was focused specifically on seminolipids. The results demonstrated a significant reduction in seminolipid levels in rats exposed to BPA. Conversely, the co‐administration of EOMC at various concentrations, as well as Vit E, showed protective effects, preserving seminolipid concentrations.

Additionally, BPA exposure led to a marked decrease in cardiolipin levels, a critical phospholipid for mitochondrial function and energy production, which is essential for sperm motility (Ren et al., 2019). The decline in cardiolipin is mostly the main reason for the reduced motility observed in BPA‐treated rats. However, the supplementation with EOMC or Vit E appeared to mitigate these effects, possibly by preserving membrane phospholipid composition, which may contribute to the stabilization of mitochondrial membranes against BPA‐induced damage.

Interestingly, we observed an increase in the concentrations of phosphatidylcholine (PC) and phosphatidylethanolamine (PE) in the BPA‐treated group. This suggested that BPA disrupted phosphatidylcholine biosynthesis, leading to decreased membrane PC levels and the accumulation of PE (Szlasa et al., 2020). The accumulation of misfolded proteins, often associated with chronic endoplasmic reticulum (ER) stress, may further contribute to these changes, a condition linked to carcinogenesis. In contrast, rats treated with EOMC or Vit E without BPA exposure maintained normal phosphatidylcholine levels, highlighting the importance of phospholipid homeostasis in preserving cellular functions (Shyu Jr et al., 2019).

Furthermore, glycosphingolipids, which are vital for cell survival, membrane protein regulation, and intercellular communication (D'Angelo et al., 2013), were significantly reduced in the BPA‐treated rats. However, treatment with EOMC oil or Vit E, even at a low dose (100 mg/kg), provided a protective effect, helping to maintain glycosphingolipid levels and underscoring their protective role in combating BPA‐induced membrane disruptions.

The results reveal several biological effects of treatments with Vit E or EOMC at different concentrations (50, 100, and 200 mg) on the seminiferous tubules. Simultaneously, exposure to BPA appears to impair spermatogenesis by inducing hypospermiogenesis. The analysis highlights both the protective potential of these treatments and their limitations under different conditions.

Vit E is well known for its antioxidant properties, which protect cells from oxidative damage. In this study, the observation of “coagulative aspects” in the seminiferous tubules treated with Vit E suggests that Vitamin E may enhance cell integrity and promote cohesion between spermatids and other germ cells. These findings indicate that vitamin E helps limit the negative impact of BPA by preserving the structure of the tubules, despite the occasional presence of immature cells. However, even with Vit E treatment, BPA exposure still results in some disruption, as evidenced by the presence of immature cells in the lumen. This suggests that although Vit E reduces the toxic effects of BPA on spermatogenesis, it cannot entirely prevent them.

EOMC was co‐administered with BPA at increasing doses (50, 100, and 200 mg), and their effects on spermatogenesis were assessed. At a low dose of 50 mg, mild hypospermiogenesis was observed, indicating a partial reduction in normal sperm formation. This suggests that at this concentration, EOMC provides moderate protection against BPA‐induced damage. However, at 100 mg, the hypospermiogenesis became more pronounced, suggesting that this intermediate dose may not be sufficient to counteract the toxic effects of BPA completely (D'Angelo et al., 2013). It is also possible that the oils exhibit a paradoxical or slightly toxic effect at this concentration. At 200 mg, the presence of a marked number of immature sperm in the lumen of some tubules suggests a stronger protective effect, but one that may result in the premature release of germ cells. This response could reflect an adaptive mechanism where cell proliferation is stimulated, even if spermatogenesis remains incomplete or compromised.

The toxic impact of BPA alone was also confirmed in this study. BPA induced hypospermiogenesis in some tubules, likely by interfering with testosterone production or triggering oxidative stress in germ cells. This confirms the well‐known detrimental effects of BPA on male reproductive health.

When comparing the effects of the two treatments, it becomes evident that Vit E provides better protection of tubule structure by reducing the presence of immature sperm, suggesting that it exerts a more targeted antioxidant effect against BPA‐induced stress (D'Angelo et al., 2013). In contrast, the effects of EOMC appear to be dose‐dependent. At the highest dose tested (200 mg), EOMC may offer greater protection, but it also increased the fraction of immature sperm, which could indicate either an adaptive response or mild toxicity. Conversely, the lowest dose tested (50 mg/kg) offers only moderate protection and was insufficient to completely mitigate the disruptions caused by BPA exposure.

In conclusion, both Vitamin E and *Myrtus communis* essential oil demonstrated protective effects against BPA‐induced reproductive toxicity in male rats. While Vit E effectively preserved the integrity of the seminiferous tubules, EOMC showed a dose‐dependent efficacy, with the 200 mg/kg dose offering the most pronounced protective effects. EOMC not only improved sperm quality and testicular histology but also helped restore reproductive hormone levels, maintain body weight, and preserve the phospholipid composition of sperm membranes. However, the potential for toxicity at higher concentrations underlines the importance of optimizing the dosage. These findings highlight the therapeutic potential of EOMC as a natural agent for mitigating the adverse effects of environmental endocrine disruptors, while also reinforcing the role of Vit E as a reference antioxidant.

## FUNDING INFORMATION

This research did not receive any specific grant from funding agencies in the public, commercial, or non‐profit sectors. This work was supported by the Higher Institute of Biotechnology of Beja, University of Jendouba, Tunisia, through the provision of laboratory facilities and materials.

## CONFLICT OF INTEREST STATEMENT

The authors declare no potential conflict of interest.

## ETHICS STATEMENT

All procedures on animals in this study were compiled with the National Institutes of Health recommendations for the use and care of animals.

## Data Availability

All data generated or analyzed during this study are included in this published article.
